# Rehabilitation Service Assessment and Workforce Capacity Building in Albania—A Civil Society Approach

**DOI:** 10.3390/ijerph17197300

**Published:** 2020-10-06

**Authors:** Boya Nugraha, Klejda Tani, Christoph Gutenbrunner

**Affiliations:** 1 Department of Rehabilitation Medicine, Hannover Medical School, 30625 Hannover, Germany; gutenbrunner.christoph@mh-hannover.de; 2 Faculty of Technical Medical Sciences, University of Medicine Tirana, AL1005 Tiranë, Albania; klejdatani@gmail.com

**Keywords:** rehabilitation service, rehabilitation workforce, rehabilitation assessment, health system, capacity building

## Abstract

Background: Rehabilitation is an important health strategy. Due to the lack of rehabilitation professionals (e.g., no physical and rehabilitation medicine, occupational therapist, and others) and lack of rehabilitation services (e.g., no multi-profession rehabilitation services in hospital, no post-acute rehabilitation services, no community-based rehabilitation services), the need to strengthen rehabilitation in Albania was pronounced. Therefore, this project aimed at rehabilitation service assessment and workforce capacity building in Albania. Methods: The World Health Organization’s Template for Rehabilitation Information Collection was used to collect available data related to rehabilitation services. Additionally, two site visits to different rehabilitation centers including interviews with relevant stakeholders were performed. A stakeholders’ workshop to prioritize recommendations was also performed before finalizing the report. Results: In Albania, rehabilitation service delivery, rehabilitation workforces, and financing in rehabilitation need to be strengthened. Conclusions: The project achieved the intended objectives. Additionally progress has been occurring in the development and implementation of the Physical and Rehabilitation Medicine specialization at the University of Medicine, Tirana.

## 1. Introduction

Rehabilitation is a health strategy that enables people experiencing disability to achieve and maintain optimal functioning [1]. From a health policy perspective, Stucki et al. highlighted that rehabilitation is the most relevant health strategy of the 21st century [2]. Rehabilitation is an integral part of universal health coverage (UHC) [3] and therefore should be available for every person in need. Furthermore, the UN Convention on the Rights of Persons with Disabilities (UNCRPD) [4] urges countries to provide rehabilitation services.

The World Report on Disability (WRD) [5] has shown, based on statistical data, that in many countries around the world, rehabilitation is not available or has a low standard; and in many others, not everyone in need has access to such services. The same is the case for assistive devices. Consequently, in 2014, the World Health Assembly adopted a Global Disability Action Plan “better health for all people with disabilities” (GDAP) [6] that has three objectives: “(1) to remove barriers and improve access to health services and programs, (2) to strengthen and extend rehabilitation, habilitation, assistive technology, assistance and support services, and community-based rehabilitation, and (3) to strengthen collection of relevant and internationally comparable data on disability and support research on disability and related services”.

Responding to the GDAP, several activities have been initiated in different countries including Mongolia [7], Malaysia [8], and Indonesia [9]. Additionally, Khan et al. also analyzed the challenges and barriers for the implementation of GDAP in low and middle-income countries in Asia and Africa [10]. Other initiatives include the International Spinal Cord Injury Survey that has been performed in more than 20 countries worldwide [11], with the latter survey was a learning health system initiative on how health systems respond to the rehabilitation needs of spinal cord injury patients as a model.

In 2017, the World Health Organization (WHO) started the Initiative “Rehabilitation 2030—a call for action” [12] and published Recommendations for the Implementation of Rehabilitation into Health Systems [13]. One main goal of the Rehabilitation 2030 initiative is to support countries in analyzing the situation of rehabilitation services within the national health systems and to develop implementation plans. For this purpose, standardized assessment methods have been developed by the WHO [14]. One mandatory precondition for such projects is the request and commitment from the government of the respective country. However, according to the Albanian project partners (the Faculty of Technical Medical Sciences of Tirana University of Medicine), such a request has not been made. In this situation, the civil society, which was represented by the Faculty of Technical Medical Sciences of Tirana University of Medicine, took the initiative to systematically analyze the rehabilitation system in Albania with the main focus on rehabilitation service provision and rehabilitation workforce capacity. This project has been realized in partnership with the Department of Rehabilitation Medicine at Hannover Medical School, Hannover, Germany.

This project aimed at rehabilitation service assessment and workforce capacity building in Albania based on existing data and compared with the standard given in the recommendation by the World Health Organization [5,6,13]. This includes information on the legal background of rehabilitation in the country as well as other relevant regulations (including payment). From this information, proposals for implementation of rehabilitation services and rehabilitation workforce capacity building should be developed (rehabilitation service implementation plan). Additionally, the project aimed at generating awareness of decision makers, other stakeholders, and the general public on the need and perspectives of strengthening rehabilitation in Albania.

## 2. Materials and Methods

In this study, a civil society approach was performed, it means that this study was done neither by nongovernmental nor commercial for profit actors [15]. In this project, two academic institutions, the Faculty of Technical Medical Sciences of Tirana University of Medicine and the Department of Rehabilitation Medicine at Hannover Medical School, Hannover, Germany as civil societies collaborated together. The research plan was presented to the Ethic Committee of Hannover Medical School. The chair of the committee stated that the study did not require ethical approval as no personal data were collected within the project, as shown in supplementary.

### 2.1. Project Phases

Based on previous experiences [16,17,18,19,20], the project consisted of five phases:

**Phase 1**: Collecting general information about the country (population, economic situation, health expenditure, health system, epidemiological data, and the availability of rehabilitation services including rehabilitation workforce. This information was covered and structured in the Template for Rehabilitation Information Collection (TRIC) [14]. Aside from information from open sources such as the WHO website, specific publications by government, statistics bureaus or universities in the country about the topic were also used. This data collection was performed by the Albanian project partners.

Missing information was added by asking to the representative institutions who work in the field.

**Phase 2**: During one week of site visits (26–28 June 2019) in Tirana and Durres, interviews with relevant stakeholders were undertaken (both as single interviews and group discussions). Additionally, a number of rehabilitation services were visited.

The stakeholders interviewed and the institutions visited were selected by the authors who at the same time represented the project partners (University of Tirana, Medical Technical Faculty and Hannover Medical School, Department of Rehabilitation Medicine). Selection criteria were set to cover as many organizations and institutions as possible to obtain the most relevant information as necessary to fill-in the TRIC tool and get the best insights into real-life service delivery. These are representatives of the organizations of rehabilitation professionals, organizations of persons with disability, rehabilitation units in acute, post-acute, and long-term care, special rehabilitation centers for children with intellectual and developmental disabilities, academic institutions (in this case representatives from Tirana University), and government officials. All stakeholders participated voluntarily. No financial or other compensation was given, neither to participating stakeholders nor to the project team, however, travel costs and accommodation during site visits of the foreign project partners were compensated by both of the universities.

All interviews were started with open-ended questions. After explaining the project goals and methodology, the interview partners were asked to describe the situation from their own perspectives and expertise. Follow-up questions were asked on the background and experience of the interviewers and aimed at a deeper insight into a better understanding of the situation (including mirroring the responses). Last but not least, missing technical information was clarified like number of staff, referral rules, and financial aspects. The results of the interviews and visits were documented in the report and were the basis of the stakeholder prioritization workshop. The project team discussed all information and checked for consistency and quality of information. If necessary, missing information was gathered post-hoc.

**Phase 3**: The results of phases 1 and 2 were discussed among the project partners and summarized in a draft report (in English). This report also contains recommendations for actions and proposals for some additional assessment and concrete implementation projects.

**Phase 4**: During a second visit in the country (30–31 January 2020), missing information was collected and relevant stakeholders who could not be seen in the first visit (phase 2) were interviewed here. A workshop with relevant stakeholders was organized to discuss and prioritize the draft recommendations and projects.

Prioritizing recommendations were done by the following methodology. At the beginning of the stakeholder workshop, the participants (*n* = 17) were informed about the goals of the project and the project methodology. Each participant received a list of recommendations as developed during the projects. Every recommendation was presented by the authors and discussed with the participants. This list contained a free column where the participants could give a score for each single recommendation. The score ranged from 1 = very high; 2 = high; 3 = middle; 4 = low; and 5 = very low. After consensus for every single recommendation was reached, every participant filled in their priority score for each recommendation. At the end of the workshop, the scoring sheets were collected and the mean scores were calculated. Additionally, a priority scale was applied by authors with the following algorithm: 1.00 to 1.49 = high priority; 1.50 to 1.99 = moderate priority, and over 2.00 = low priority.

**Phase 5**: With the results of phase 4, a final report was developed in English and later translated into thee Albanian language. The information of the report will be disseminated to all relevant stakeholders in the country.

### 2.2. Stakeholders in each phase

Site visits and interviews were conducted at different organizations or health services including: the Department of Rehabilitation at Faculty of Technical Medical Sciences, University of Medicine (phases 1, 2, 3, 4, and 5; one representative at stakeholder workshop (SW)); PT service in the Department of Neurosurgery University Hospital “Mother Teresa” (phases 2 and 4; two representatives at SW); Association of People with Para-/Tetraplegia (phases 2 and 4; one representative at SW); Ministry of Health and Social Protection (phase 2 and 4; one representative at SW); University of Medicine, Tirana (phases 2 and 4; one representative at SW); Regional Hospital of Durres (phase 2; one representative at SW); PT service in the University Hospital of Trauma (phases 2 and 4; one representative at SW); PT service in the Department of Rheumatology, University Hospital “Mother Teresa” (phases 2 and 4; one representative at SW); National Center for Child Growth, Development and Rehabilitation, Tirana (phases 2 and 4; one representative at SW); Central Polyclinic of Tirana (phase 2; no representatives at SW); Faculty of Technical Medical Sciences, University of Medicine, Tirana (phases 2 and 4; three representatives at SW); Regional Director of Tirana, MOHSP, Tirana, Albania (phases 2 and 4; one representative at SW); Department of Physiotherapy in the University Hospital “Mother Teresa” (phase 4; one representative at SW); Physiotherapy Association, Albania (phases 2 and 4; one representative at SW); and the Association of Speech and Language Therapy, Albania (phases 2 and 4, one representative at SW).

## 3. Results

### 3.1. Epidemiological and Health System Data

Albania has a population of 2,862,427. The sex distribution in Albania consists of 49.95% male and 50.05% female, with life expectancy of 78.34 years. The prevalence of disability in Albania is 6.2% [21]. Meanwhile, the prevalence of non-communicable disease (NCD) such as cancer, diabetes, cardiovascular, and respiratory diseases are 4.2%, 2.5%, 30.0%, and 1.3%, respectively [21]. The number of cases of stroke is 4400 per year. The incidence of sensory illness recorded between 2010 and 2017 was 348,995. The prevalence of dementia is 9.6 per 1000 population [21] and the prevalence of mental health conditions is 8.3% [21]. No information is available with regard to the prevalence of intellectual and developmental disabilities and neurological conditions in children. The prevalence of overall musculoskeletal conditions is 9.75% and the incidence of hip fractures totaled 301 cases in 2016 [21].

The Ministry of Health and Social Protection (MOHSP) is responsible for rehabilitation in the country. However, rehabilitation is not included in health policy and legislation frameworks yet. Rehabilitation is also not included in the national health strategic plan and is only included within mental health areas, particularly in pediatrics.

The MOHSP is responsible for health related issues including rehabilitation. Total health expenditure in 2014 in Albania was 5.9% of GDP [22], which is about 615 USD per capita [22]. From this amount, there is no specific budget allocation for rehabilitation, however, there is an allocated budget for assistive devices, although without a specific amount.

In Albania, there is a financing mechanism for people with disabilities, which is called *Aftësi të Kufizuara* (PAK). It has a defined budget of 160 million USD per year. However, it is not dedicated to rehabilitation services, only for living expenses and assistance payments.

### 3.2. Rehabilitation Workforce

In Albania, physiotherapists and psychologists are available at primary, secondary, and tertiary levels of health care such as physiotherapy or psychology practice, secondary hospital, and tertiary hospital, respectively. Speech and language therapists are available at secondary and tertiary hospitals.

Table 1 demonstrates the data of rehabilitation professions in Albania. As aforementioned, there are only three different professions available in Albania, namely physiotherapists (PT), speech and language therapists (SLT), and psychologists. The numbers of these professions are 1108 for PTs, 136 for SLTs, and more than 200 for psychologists. A bachelor degree is the minimum requirement for these professions to work professionally. Nine institutions offer a physiotherapy program, two offer speech and language therapy programs, and seven offer psychology. Some of these institutions provide bachelor, master, and doctorate programs. Annually, 450 physiotherapists, 60 speech and language therapists, and 400 psychologists graduate from these institutions. These professions have national associations that are also affiliated to international or world associations. The licensing of PT and SLT is under the order of nurse; psychologists fall under the order of psychologists. Although there is no physical and rehabilitation medicine (PRM) physician and occupational therapist (OT) in the country, some medical doctors had attended a one-year program of physical therapy in 2009 that was held with the support of Handicap International with some PRM specialists from France. Therefore, there is professional registration/licensing for PRM in Albania, which is under the order of doctors.

### 3.3. Rehabilitation Service Delivery

In Albania, there is no comprehensive/specialized rehabilitation (inpatients) center for complex rehabilitation needs. Some hospitals provide rehabilitation services in secondary and tertiary hospitals, which deliver both in- and outpatients. In some children’s hospitals, beds for rehabilitation services are available.

Primary care rehabilitation services are available including in private clinics (PT services), but the patients should pay their own expenses. This type of rehabilitation service is available only in urban areas and big cities in the country. A service provision package of primary health care is available, but rehabilitation is not included in the package.

Realizing the importance of rehabilitation, hospitals and polyclinics organize self-financing mechanisms for rehabilitation. Rehabilitation services are available at some departments in the University Hospital of Mother Theresa Tirana such as infectious diseases, neurology, trauma, and pediatrics; the rehabilitation department at the University Hospital of Trauma; ambulatory rehabilitation in policlinics; and some other rehabilitation departments in other hospitals in the big cities of Albania. Meanwhile, rehabilitation for children with intellectual and developmental disabilities is covered by the MOHSP.

Rehabilitation services are not available in acute care. In sub-acute care, rehabilitation services are available at the University Hospital “Mother Theresa” Tirana, Department of Pediatrics, Neurology and Rheumatology (inpatients and outpatients); university hospital of trauma (inpatients and outpatients); and regional hospitals of other cities (inpatients and outpatients). Long-term rehabilitation services are available only for patients with mental health, for example, the Children’s Development Center-Pellumbat in Tirana.

Rehabilitation services for children are available at some hospitals including the Department of Pediatrics of the University Hospital “Mother Theresa” where both in- and outpatients services are delivered, and the National Center for Child Growth, Development, and Rehabilitation, Tirana for inpatients and outpatients. However, there are no mechanisms within the health services that support early identification/detection and referral for children with developmental delays and disabilities.

### 3.4. Recommendations and Projects

#### Recommendation

Based on the results of the situation analysis and site visits, a list of recommendations were proposed. This list was based on the recommendations in the GDAP [6], WRD [5], and Rehabilitation in Health System [13].This list was prioritized at the stakeholders’ workshop. Below are the results:

- High priority (mean score: 1.00–1.49)
  - PRM should be implemented as an independent medical specialty in Albania (mean score: 1.33)
  - Rehabilitation as a health strategy must be implemented in all phases (*acute, post-acute, long-term care including Community Based Rehabilitation*) and levels of medical care (*primary, secondary, and tertiary care*), and should include early detection and intervention (mean score: 1.08)
  - Work toward a participation model for the inclusion of persons with disabilities to be used as a basis of planning and decision making at all relevant fields of political actions such as health, education, labor, justice, social welfare as well as the planning of public and private buildings and public transport system. Such a model should replace the charity model of rehabilitation services and social compensation programs that nowadays is predominately used in Albania (mean score: 1.17)
  - At a scientific level and as one first step in evaluating the life situation and rehabilitation provision of persons with disabilities, it is recommended to establish an Albanian section of the International Spinal Cord Injury (InSCI) survey [11,23] (mean score: 1.08).
  - As above-mentioned, the implementation of rehabilitation medicine topics in undergraduate medical training can be a first step to increasing knowledge in rehabilitation in the medical community. In parallel, the implementation of PRM specialist training at the University of Medicine Medical Faculty in Tirana can be done (mean score: 1.42).
  - To further develop the skills and competencies of already trained physiotherapists, it is recommended to develop standards for physiotherapy for different conditions and services based on the best available evidence (mean score: 1.09).
  - Develop standards for staffing and technical equipment for specific rehabilitation services (mean score: 1.33)
  - To learn about the existing rehabilitation services in the country, a rehabilitation service survey in the country that is based on the International Classification of Service Organization in Rehabilitation (ICSO-R) can be implemented [24,25] (mean score: 1.25).
  - As some of the above projects will require financial support, it is important to develop a funding strategy for such projects (*Charity funding, use existing national research funds, apply for European Union funds*) (mean score: 1.42).
- Moderate priority (mean score: 1.50–1.99)
  - Ensure that rehabilitation is fully integrated in the health system and rehabilitation services are given a priority together with the other element of universal health coverage (UHC) (mean score: 1.50).
  - Ensure that the International Classification of Functioning, Disability and Health (ICF) is used for assessment and follow-up of disability as well as health care planning. The UN definition of disability as an interaction of an individual with a health condition and the environment should be implemented at all levels of decision making (mean score: 1.92).
  - Asses systematically the prevalence of disability in Albania and estimate the needs for rehabilitation using the Model Disability Survey (MDS) of the WHO [26] or another appropriate method of needs assessments (mean score: 1.68).
  - Increase the understanding and awareness of disability in Albanian society (e.g., by public relations campaigns) and ensure that disability and rehabilitation topics are taught in the undergraduate training of medical doctors, health professionals, and social workers as well as teachers, architects, city planners, and others (mean score: 1.75).
  - The disability health policy should be based on the WHO’s modern understanding of disability (according to the ICF model) as an interaction of a person with a health condition and the environment (mean score: 1.75).
  - Rehabilitation services should be conceptualized and implemented according to the international standards including qualification of rehabilitation professionals, team structures, and standards for technical equipment as well as assistive devices (mean score: 1.75).
  - Primary care physicians should receive continuing education training in basic rehabilitation skills (mean score: 1.92).
  - Training program and accreditation rules for occupational therapy should be developed and implemented (e.g., within the Faculty of Medical Technical Sciences) (Mean score: 1.50).
  - Add clinical rehabilitation modules to the training programs for logopedists (SLT) and their numbers should be increased (mean score: 1.75).
  - Training program and accreditation rules for prosthetics and orthotics should be developed and implemented (mean score: 1.50).
  - A CME program for therapists (and a system for quality assurance (including audits) should be developed and implemented stepwise (mean score: 1.67).
  - Implementation of occupational therapy in University of Medicine Faculty of Medical Technical Sciences (mean score: 1.67)
  - Tele-health/tele-rehabilitation programs should be developed by a scientific expert group (mean score: 1.75)
- Low priority (score ≥ 2.00)
  - Training courses in rehabilitation for nurses are recommended, particularly for nurses who work in acute rehabilitation and highly specialized rehabilitation centers) (mean score: 3.00).

## 4. Discussion

This project aimed at an estimation of the need for rehabilitation in Albania and an analysis of the existing rehabilitation services. This was not the National Strategic Plan for Rehabilitation according to the standards of the WHO [14] that starts from the macro level of the health care system and aims at implementing rehabilitation into the National Health Strategic Plan. Such projects must be initiated by the government and performed by the WHO (together with partner organizations). However, there are overlaps of both approaches and—as far as possible—elements of the WHO framework were used. As there were no initiative actions from the government, this project was initiated by the Faculty of Technical Medical Sciences of Tirana University of Medicine in partnership with the Department of Rehabilitation Medicine, Hannover Medical School, Hannover, Germany.

As aforementioned in the results, the need to strengthen rehabilitation services in Albania is pronounced. It includes the rehabilitation service provision, rehabilitation workforces, and financing for rehabilitation services. Realizing the importance of rehabilitation, some hospitals and polyclinics are organizing self-financing mechanisms for rehabilitation.

Recommendations were derived either from objective data in relation to internationally accepted standards for rehabilitation service provision (mainly based on the GDAP, WRD, and Recommendation of Rehabilitation in Health System), this mainly includes implementation of rehabilitation professions not yet existing in Albania (e.g., PRM, OT, P&O) and establishment of rehabilitation services that do not exist (e.g., early rehabilitation, post-acute rehabilitation centers, community based rehabilitation services). Other recommendations are to respond to qualitative information such as the attitude of the majority of the population with regard to people with disabilities and understanding of full participation, the lack of understanding in the need of persons with disability among health professionals, and the lack of quality controls and CME for rehabilitation practitioners.

During the first site visit, one of the discussions was with the university and faculties that concluded that having education in the specialization of PRM at the University of Medicine Tirana was one of the priorities. Additionally, the need to include rehabilitation in the undergraduate module is also very high. Therefore, the curriculum for PRM specialization and the module for rehabilitation in an undergraduate degree were proposed to the University of Medicine Tirana. It was also discussed of how to get support for implementing the PRM specialization from the international PRM community, particularly from the *Union Européenne des Médecins Specialistes* (UEMS)-PRM Section. To implement other health related professions such as OT, and P&O, the authors also recommend that the University of Medicine Tirana collaborate with other international organizations such as the World Federation of Occupational Therapy and International Society of Prosthetists and Orthotists, respectively.

Due to the lack of the examples of strengthening rehabilitation at the health system level, the methodology of this project was based on our previous experiences in establishing a National Disability and Rehabilitation Action Plan in different countries [17,18,19]. A comparison of the methodology can be seen in Table 2.

Although this study addressed Albania, it can be applied as learning experience for other countries worldwide where governments have not initiated strengthening rehabilitation and the civil society can initiate such projects. Of course, it should be done systematically and with sound methodology. Therefore, our approach for Albania was to use the WHO’s tool and previous experiences [18,19,27]. This study also supports increasing the awareness of the needs and reports of research in the health system and policy research in specific countries [28]. Despite this project not officially being supported by the government, the commitment of people in this country has led to the high level of success of this project.

Limitations. In spite of the achieved of intended objectives of this project, some limitations can also be observed. These include: (1) the site visits were only performed in two cities, Tirana and Durres; (2) during the stakeholders workshop, there was no equal representation of different health professionals in rehabilitation, this was due to the lack or even non-existing rehabilitation professionals in the country, such as PRM, OT, P&O and rehabilitation nurse. We tried to compensate it optimally by involving representatives from different institutions who worked in the field Rehabilitation. 

## 5. Conclusions

Although there was lacking official support from the government, this project achieved the intended objectives (e.g., the development and the implementation of curricular for PRM specialization at the University of Medicine Tirana has been started). This civil society approach can be performed as a model and alternative for such purpose and might lead to more objective results. The authors hope that the results and recommendations in the final report will represent a milestone in the improvement of rehabilitation services in Albania.

## Figures and Tables

**Table 1:** Data of rehabilitation professions in Albania.

Rehabilitation Professionals	Total Numbers	Distribution Urban Rural; % in Primary, Secondary, Tertiary	Minimum Educational Requirement for Entry into the Workforce	Which of the Following are Offered in the Country	Number of Education Institutions Offering Courses	Number of National Graduates Each Year	Is there Professional Registration/Licensing?	Is There a Profession Association
**PT**	1108	No data	Bachelor	Bachelor, master, doctorate	9	450	Yes	Yes
**OT**	None	None	None	None	None	None	None	None
**SLT**	136	No data	Bachelor	Bachelor, master, doctorate	2	60	Yes	Yes
**P&O**	None	None	None	None	None	None	None	None
**PRM physicians**	None	None	None	None	None	None	Yes	None
**Rehabilitation Nurses**	None	None	None	None	None	None	Yes	None
**Psychologists**	>200*	No data	Bachelor	Bachelor, master, doctorate	7	400	Yes	Yes
**Other rehabilitation cadre**	None	None	None	None	None	None	None	None

PT: Physiotherapist; OT: Occupational Therapist; SLT: Speech and Language Therapist; P&O: Prosthetist and Orthotist; PRM: Physical and Rehabilitation Medicine; * Only with licenses in clinical psychologist.

**Table 2:** Comparison of rehabilitation assessment in Albania, Ukraine, Democratic People’s Republic of Korea (DPRK), and Egypt.

		Country			
		Albania	Ukraine [19]	DPRK [20]	Egypt [18]
Year of Project		2019–2020	2015	2016–2017	2015
**Methodology**	Initiated by	Civil Society (*University*)	Government (*Ministry of Heath*)	Government	Government (*Ministry of Heath*)
	Data collection tool	TRIC	RSAT	RSAT	RSAT
	Site visit to the country and interview	2	2	No site visit	2
	Additional working meetings	Video conferences	-	Two visits of DPRK team to Germany	Multiple correction loops with WHO country office via email
	Team	PT, PRM, rehabilitation researcher	WHO, PRMs, neurorehabilitation	PRM, representative of persons with disability organization (KFPD), neurorehab	WHO, PRM
	Stakeholders	Rehabilitation professional organizations (PT, ST), a representative of MOSP, rehabilitation service hospitals	Rehabilitation professional organizations (PT, ST), representative of MoH, MoSW rehabilitation service hospitals	Representative of persons disability organization (KFPD) and PRM from rehabilitation hospital	Rehabilitation professional organizations (PRM, PT) representative of MoH, MoSW, rehabilitation service hospitals
**Priority Recommendations (*categories*)**	Develop national rehabilitation action plan	-	Yes	Yes	Yes
	Strengthen inter-ministerial communication	-	Yes	Yes	Yes
	Translation and implementation of ICF	Yes	Yes (has been started)	Yes	Yes
	Implement rehabilitation professions	PRM, OT, P&O	PRM, OT, for PT transition to international standards	Transition to international standards	N
	Expand rehabilitation services	Acute, post-acute services, and CBR	Acute, post-acute services and CBR	Acute, post-acute services, and CBR	Acute, post-acute services, and CBR
	Improve delivery of Assistive Technology	Yes	Link with rehabilitation services	Yes	Yes
	Strengthen rights of persons with disability	Yes	Yes	Yes	Yes
**Priority projects**	Development or adaptation of curricula for rehabilitation professions	Yes	Yes	Yes	Yes
	Perform national disability survey	Yes (*i.e., start with InSCI project*)	Yes	Yes	Yes
	React to specific situation	-	Armed conflict in eastern Ukraine	Collaborate with international partners	Services for persons without health insurance

Note: DPRK: Democratic People’s Republic of Korea; TRIC: Template for Rehabilitation information Collection; RSAT: Rehabilitation Service Assessment Tool; InSCI: International survey on Spinal Cord Injury; CBR: Community-based rehabilitation; MOSP: Ministry of Social Protection; MoSW: Ministry of Social Welfare; MoH: Ministry of Health; KFPD: Korean Federation for People with Disability.

## Supplementary Materials

The following are available online at https://www.mdpi.com/1660-4601/17/19/7300/s1.
