# Supplementary material for: Rehabilitation Service Assessment and Workforce Capacity Building in Albania—A Civil Society Approach
Source: Int J Environ Res Public Health. 2020 Oct 6;17(19):7300. doi: 10.3390/ijerph17197300 (PMC7579078; doi:10.3390/ijerph17197300)
Supplement: Supplementary file 1 [file ijerph-17-07300-s001.pdf]

Medizinische Hochschule Hannover

Ethikkommission
Vorsitzender:
Prof. Dr. Stefan Engeli

Sekretariat:

Marion Lange
Telefon: 0511 532-3443

Liane Höft
Telefon: 0511 532-9812

Fax: 0511 532-16 3443
ethikkommission@mh-hannover.de

Carl-Neuberg-Straße 1
30625 Hannover
Telefon: 0511 532-0
www.mh-hannover.de

MHH Ethikkommission OE 9515
30623 Hannover

Manuscript "Rehabilitation Service Assessment and Workforce Capacity Building in Albania – A Civil Society Approach"

To the Editor

Prof. Gutenbrunner addressed me with a question forwarded from the *International Journal of Environmental Research and Public Health* regarding his above mentioned manuscript. As Chairman of the Ethics Committee of Hannover Medical School, I want to clarify that this project is not a study in the general medical meaning, and therefore, based on the German Medical Association's professional code of conduct, no ethic committee approval is necessary. However, having checked the content of the manuscript, I do not see any concern from the point of view of Medical Ethics.

With kind regards

Prof. Stefan Engeli
Chairman
Ethic Committee of Hannover Medical School
